# The counter regulatory response induced by CpG oligonucleotides prevents bleomycin induced pneumopathy

**DOI:** 10.1186/1465-9921-13-47

**Published:** 2012-06-18

**Authors:** Takeshi Kinjo, Koji Tomaru, Diana C Haines, Dennis M Klinman

**Affiliations:** 1 Cancer and Inflammation Program, National Cancer Institute, Bldg 567, Rm 205, NCI at Frederick, Frederick, MD, 21702, USA; 2 Pathology and Histotechnology Laboratory, SAIC-Frederick, Inc. National Cancer Institute, Frederick, MD, 21702, USA

**Keywords:** Bleomycin, Pneumonitis, Fibrosis, CpG oligonucleotide

## Abstract

Bleomycin (BLM) induces life-threatening pneumonitis and pulmonary fibrosis in 20% of patients, limiting its use as a chemotherapeutic agent. Oligonucleotides expressing immunostimulatory CpG motifs (CpG ODN) stimulate cells that express Toll-like receptor 9 to initiate an inflammatory response. This short-lived inflammation is physiologically suppressed by a counter-regulatory process that peaks five days later. Using a murine model of BLM-induced lung injury, the effect of CpG ODN treatment on pulmonary inflammation, fibrosis and mortality was examined. Administering CpG ODN 5 days before BLM (so that the peak of the counter-regulatory process induced by CpG ODN coincided with BLM delivery) resulted in a dose-dependent reduction in pulmonary toxicity (p < 0.005). Delaying the initiation of therapy until the day of or after BLM administration worsened the inflammatory process, consistent with the counter-regulatory process rather than initial pro-inflammatory response being critical to CpG induced protection. The protection afforded by CpG ODN correlated with reduced leukocyte accumulation and inflammatory cytokine/chemokine production in the lungs. These changes were associated with the increased production of IL-10, a critical element of the counter-regulatory process triggered by CpG ODN, and the concomitant down-regulation of BLM-induced IL-17A and TGF-β1 (which promote pulmonary toxicity). This work represents the first example of the physiologic counter-regulation of TLR induced immune activation being harnessed to block an unrelated inflammatory response.

## Introduction

Bleomycin is effective in the chemotherapy of various cancers including squamous cell carcinomas of the head and neck, cervix, and esophagus, germ cell tumors and both Hodgkins and non-Hodgkins lymphoma [1-3]. BLM exerts its anti-tumor activity by generating oxygen and free radicals via the reduction of Fe (II) to Fe (III). These free radicals cause DNA strand breakage and cell death [4-6]. BLM is metabolized/inactivated under physiologic conditions by bleomycin hydrolase. As there is a paucity of this enzyme in the lungs, they are unusually susceptible to BLM-induced toxicity. While several pulmonary syndromes associated with BLM administration have been described, the most common dose-limiting side effect of this therapy is interstitial pneumonitis progressing to fibrosis [7-9]. Approximately 20% of patients treated with BLM develop pneumonitis, with a mortality that approaches 20% [7,10-12].

The lung cells damaged by BLM release uric acid and other factors that trigger alveolar macrophages to secrete pro-inflammatory cytokines and chemokines (including IL-1β, IL-6, KC and MIP-2). These recruit additional inflammatory cells to the lung that contribute to the development of bleomycin-induced pneumonitis (BIP) and pulmonary fibrosis [13-15]. Recent studies indicate that IL-17A, TGF-β, and IL-10 are critical regulators of BLM-induced pneumopathy. IL-17A and TGF-β synergistically promote the development of BIP whereas IL-10 down-regulates IL-17A and thus abrogates disease [16]. The importance of these factors was confirmed in animal models showing that BLM-induced lung injury was reduced in mice in which IL-17A or its receptor were deleted/blocked, while IL-10 KO mice developed more severe BLM-induced lung inflammation and fibrosis [16-19].

The unmethylated CpG motifs present in bacterial DNA stimulate cells that express Toll-like receptor 9 (TLR9). This interaction triggers a short-lived innate immune response characterized by the production of pro-inflammatory cytokines and chemokines [20-22]. The immune stimulation induced by bacterial DNA is duplicated by synthetic oligonucleotides expressing CpG motifs (CpG ODN).

Microarray identification of the genes triggered by CpG ODN showed that the inflammatory response peaked on day 1, but that >96% of these genes were suppressed by a counter-regulatory process that peaked on day 5 [23,24]. IL-10 was identified as a key mediator of this down-regulatory process, based on both bioinformatic and functional studies of CpG-induced inflammation [23,25,26]. As noted above, IL-10 also reduces BLM-induced pulmonary inflammation by down-regulating IL-17A production. This combination of findings raised the possibility that the counter-regulatory process induced by CpG ODN might be harnessed to limit BLM-dependent pneumonitis. A murine model was used to examine the effect of CpG ODN on BLM-induced morbidity and mortality, and analyze the impact of this treatment on the production and regulation of pro-inflammatory cytokines and chemokines.

## Materials and methods

### Oligodeoxynucleotides

Phosphorothioate CpG ODN 1555 (sequence: GCTAGACGTTAGCGT) and control ODN 1612 (sequence: GCTAGAGCTTAGGCT) were synthesized at the Center for Biologics Core Facility. ODNs were administered by intraperitoneal injection in all experiments. All ODNs were free of endotoxin and protein contamination.

### BLM-induced lung injury model

Female C57BL/6 mice were studied at 7-12 wk of age. BLM (Sigma-Aldrich, St. Louis, MO) in 50 μl of PBS was instilled endotracheally into the lungs of anesthetized mice using a 24-gauge catheter. All experiments were approved by the NCI Animal Care and Use Committee, and mice were monitored daily.

### Collection of bronchoalveolar lavage (BAL) fluid and cells

The trachea was cannulated with a 22-gauge catheter and BAL fluid collected as previously described [27]. BAL was centrifuged and supernatants analyzed as described below. Cell pellets were re-suspended and analyzed histologically after cytocentrifugation and Diff-Quick staining (Dade Behring, Deerfield, IL).

### *In vitro* stimulation of LN and BAL cells

A single cell suspension prepared from the draining thoracic LN and BAL was plated at 0.5 - 4 x 10^5^ cells per well in 96-well microtiter plates and stimulated with 50 ng/ml PMA and 1 μg/ml Ionomycin (Sigma-Aldrich) for 4 hr in RPMI 1640 medium supplemented with 5% FCS, 50 U/ml penicillin and 50 μg/ml streptomycin.

#### Elisa

KC, MIP-2, IL-1β, IL-6 and IL-17A levels in BAL and culture supernatants were measured by ELISA, as previously described [28]. TGF-β1 was measured by ELISA (R&D Systems).

### Evaluation of lung fibrosis

Total soluble lung collagen was detected using the Sircol collagen assay (Biocolor, Northern Ireland, UK). Lung tissue was fixed and stained with Masson’s Trichrome. The severity of lung fibrosis were evaluated by a pathologist blinded to the origin of each sample.

### RNA isolation and quantitative RT-PCR

RNA was extracted from homogenized lung using the RNeasy Mini Kit (QIAGEN, Valencia, CA) and cDNA was generated using the Reverse Transcription Kit (QIAGEN). The cDNA from each sample was used for a quantitative RT-PCR based on TaqMan chemistry using the Step One Plus real-time PCR System (Applied Biosystems). The reaction mixture contained Taqman Gene Expression Master Mix with Taqman probes for the IL-10 gene with FAM dye (Mm00439616m1) and for the GAPDH gene with VIC dye (Applied Biosystems, Foster City, CA).

### Statistical analysis

Differences between groups were assessed using a one-way ANOVA and differences in survival rate were determined using the Kaplan-Meier log-rank test. P values ≤ 0.05 were considered significant for all analyses.

## Results

### Mice pre-treated with CpG ODN survive BLM-induced lung injury

**Figure 1:**
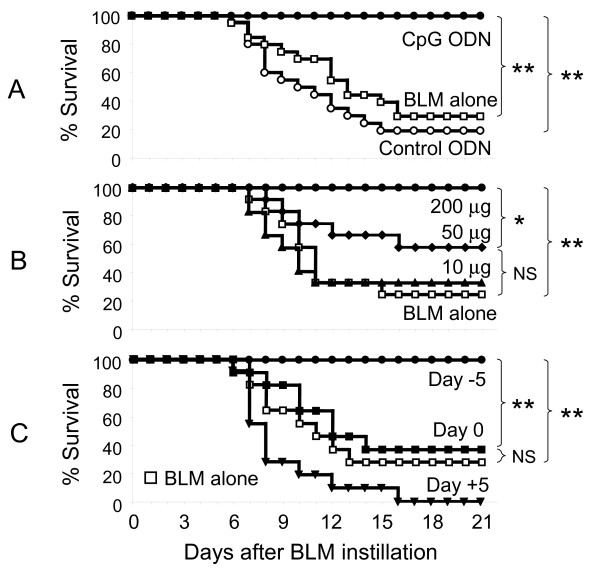
**CpG ODN protect mice from lethal BLM-induced lung injury.** 0.05 U of BLM was delivered into the lungs of C57BL/6 mice via the intra-tracheal route on day 0. A) Five days earlier, mice were injected i.p. with 200 μg of control or CpG ODN. B) Mice were treated with 0 - 200 μg of CpG ODN 5 days before BLM. C) Mice were treated with 200 μg of CpG ODN 5 days before, at the same time, or 5 days after BLM instillation. Data represent combined results from 2-3 independent experiments involving a total of 11-20 mice/group. Survival was analyzed with Kaplan-Meier statistics using the log rank test. *; p < 0.05, **; p < 0.005.

Preliminary studies established that intra-tracheal instillation of 0.05 U of BLM consistently induced severe pulmonary inflammation. The severity of the resultant pneumonitis required that 80% of the animals be euthanized within 3 wk. As CpG ODN treatment induces a short-term inflammatory response that is down-regulated under physiological conditions after 3 - 5 days, the effect of CpG ODN administration on BLM induced toxicity was evaluated. As seen in Figure 1A, lethal pulmonary inflammation was prevented by treating mice with 200 μg of CpG ODN 5 days before BLM instillation (p < 0.005). This effect was CpG specific, as control ODN had no impact on survival. CpG dependent protection was also dose dependent, with only 60% and 30% of mice surviving when treated with 50 μg and 10 μg of CpG ODN, respectively (Figure 1B). The timing of ODN delivery had a major impact on survival. Whereas CpG ODN administered 5 days before BLM challenge was highly protective, delaying treatment until the day of or five days after BLM instillation conferred no significant protection (Figure 1C). Administering CpG ODN 3 days before BLM instillation (when the counter-regulatory process was nascent) was less effective, leading to a 26% improvement in survival (p < 0.05, data not shown).

### Pre-treatment with CpG ODN reduces BLM-induced pulmonary inflammation

Previous studies showed that uric acid released by BLM-damaged lung cells trigger alveolar macrophages via the Nalp3 inflammasome signaling cascade [14]. These macrophages produce chemokines (including KC, MIP-2) and pro-inflammatory cytokines (including IL-6 and IL-1β) that recruit other inflammatory cells to the lung, culminating in pulmonary fibrosis [13-15].

**Figure 2:**
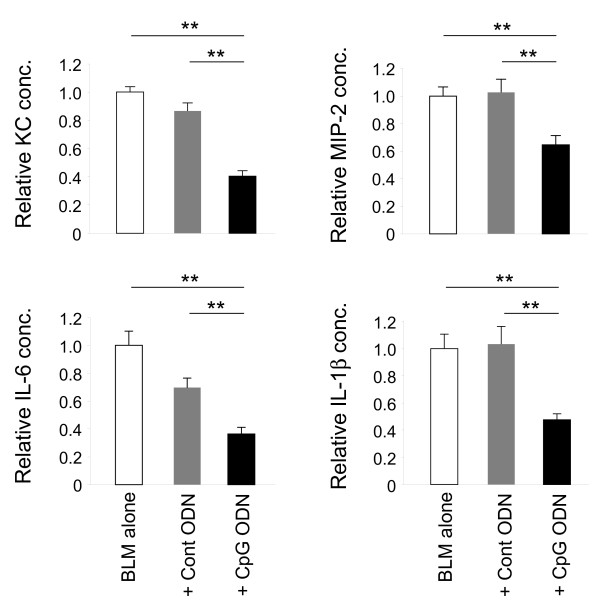
**Effect of CpG ODN on BLM-induced cytokine production.** Mice were injected i.p. with 200 μg of control or CpG ODN 5 days before 0.05 U of BLM was delivery intra-tracheally. The concentration of KC, MIP-2, IL-6 and IL-1β in BAL fluid collected 1 (KC and MIP-2) or 3 (IL-6 and IL-1β) days after BLM instillation was determined by ELISA. Results represent the average fold change + SE in cytokine levels compared to BLM alone in 2-3 independent experiments involving a total of 10-19 mice/group. **; p < 0.005.

**Figure 3:**
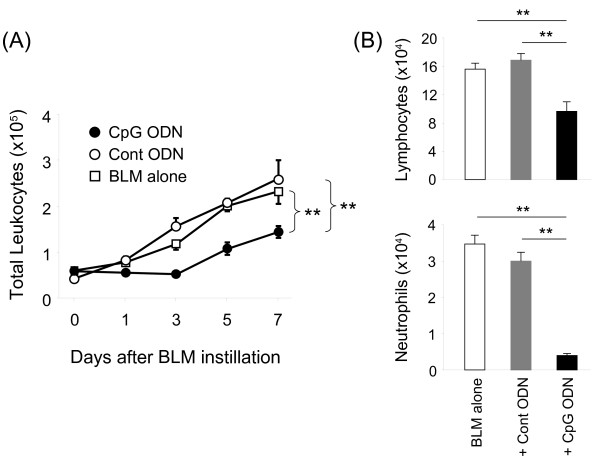
**Effect of CpG ODN on BLM-induced leukocyte accumulation in the lungs.** Mice were injected i.p. with 200 μg of control or CpG ODN 5 days before 0.05 U of BLM was delivery intra-tracheally. A) The number of leukocytes present in BAL collected 1 - 7 days after BLM instillation was determined. Statistic significance was analyzed by longitudinal regression analysis. B) Cell differentials (based on 300 leukocytes/sample) in BAL collected 5 days after BLM instillation were performed on cytocentrifuge preparations after Diff-Quick staining. Data show the mean ± SE of the combined results from 2 independent experiments involving 10 mice/group/time point. **; p < 0.005.

To evaluate the effect of CpG treatment on BLM-induced lung injury, BAL fluid was collected 1 - 7 days after BLM instillation. Consistent with previous reports, BLM triggered a significant increase in the concentration of KC and MIP-2 (peaking on day 1 and persisting through day 5) as well as IL-6 and IL-1β (peaking day 3 and persisting though day 7, Figure 2 and data not shown). Consistent with the improved survival of mice pre-treated with CpG ODN, the BAL of CpG treated mice had significantly lower levels of these factors than did untreated animals (p < 0.005, Figure 2). The accumulation of infiltrating leukocytes (most notably neutrophils) in the BAL of CpG treated mice was also significantly reduced (p < 0.005; Figure 3). By comparison, control ODN had no significant effect on leukocyte infiltration or pulmonary cytokine/chemokine levels after BLM administration.

### Pre-treatment with CpG ODN inhibits lung fibrosis

**Figure 4:**
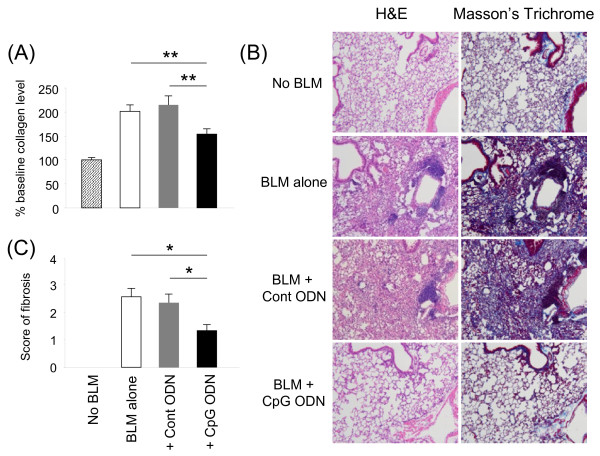
**Pretreatment with CpG ODN inhibits lung fibrosis.** Mice were injected i.p. with 200 μg of control or CpG ODN 5 days before 0.03 U of BLM or PBS (no BLM) was delivery intra-tracheally. Lungs were removed 14 days later. A) The amount of collagen was determined analytically using the Sircol collagen assay. Data show the percent change in the amount of collagen in experimental groups vs mice that did not receive BLM. This was calculated by combining results from 3 independent experiments involving 10-15 mice/group. B) Representative histologic sections from these lungs. C) Lung tissue was fixed and stained with Masson’s Trichrome. The severity of lung fibrosis were graded as (0) none, (1) minimal, (2) mild, (3) moderate and (4) severe. Data shows the mean + SE of the combined results from 2 independent experiments involving 6-7 mice/group. *; p < 0.05, **; p < 0.005.

BLM causes acute lung injury that evolves into chronic pulmonary fibrosis [7-9]. The accumulation of collagen in the lung is a characteristic marker of the magnitude of this effect. To examine whether CpG ODN impacts chronic pulmonary fibrosis, a sub-lethal dose of BLM was administered (the lower dose was needed to insure the survival of control groups for comparison purposes). BLM alone induced a 100% increase in lung collagen levels within 2 wk of administration. This effect was significantly reduced by pre-treatment with CpG ODN (Figure 4A). No reduction in fibrosis was observed in animals treated with control ODN.

Histologic analysis of lung tissue from these animals confirmed that BLM caused the development of patchy, multi-focal inflammation and markedly increased collagen deposition (Figure 4B). These histologic changes were unaffected by the administration of control ODN or PBS, whereas CpG ODN treatment resulted in significantly less severe inflammatory changes and lower levels of collagen deposition (Figure 4B, C).

### Effect of CpG ODN on IL-10, IL-17 and TGF-β expression

**Figure 5:**
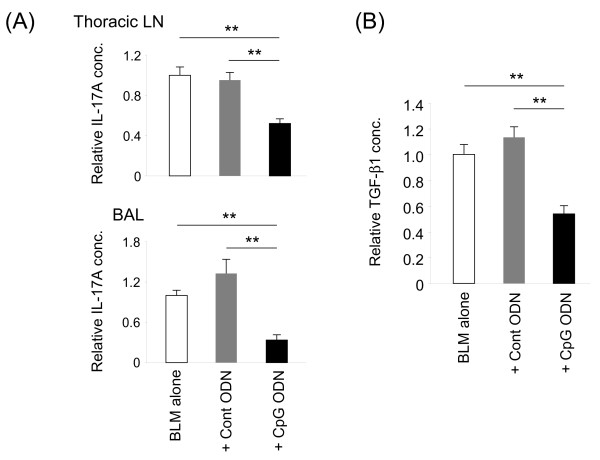
**CpG ODN treatment decreases IL-17A and TGF-β1 production.** Mice were treated as described in Figure 2. A) Cells were isolated from the BAL and draining thoracic lymph nodes 3 days after BLM instillation. These cells were stimulated *in vitro* with 50 ng/ml PMA plus 1 μg/ml ionomycin for 4 hours. The concentration of IL-17A in culture supernatants was determined by ELISA. B) The concentration of TGF-β1 in BAL fluid collected 3 days after BLM instillation was determined by ELISA. Data show the change in concentration of IL-17A and TGF-β1 (avg + SE) as a percentage of the BLM treated group alone from 2-3 independent experiments involving 9-14 mice/group. **; p < 0.005.

Recent reports show that IL-17A plays a critical role in BLM-induced lung injury, and that it interacts synergistically with TGF-β to amplify the development of fibrosis [16-18]. The effect of CpG ODN on IL-17A and TGF-β1 levels was therefore examined. As expected, the concentration of both factors rose significantly in the BAL of mice receiving BLM (Figure 5 and data not shown). Pre-treatment with CpG (but not control) ODN significantly reduced the concentration of both cytokines (Figure 5). This effect was attributable to a decrease in the production (rather than accelerated catabolism) of these factors, as lymphocytes isolated from the draining LN and BAL of CpG treated animals produced significantly less IL-17A when re-stimulated with PMA and ionomycin *in vitro* (Figure 5A).

**Figure 6:**
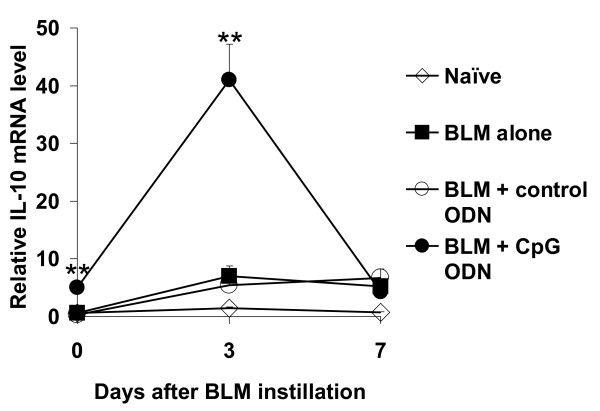
**CpG ODN treatment induces the expression of IL-10.** Mice were injected i.p. with 200 μg of control or CpG ODN 5 days before 0.05 U of BLM was delivery intra-tracheally. Mice were sacrificed immediately before BLM (day 0 time point) or 3 and 7 days after BLM. IL-10 mRNA expression in the lung was measured by quantitative RT-PCR. Data represent the mean + SE of combined results from 2 independent experiments involving 8-10 mice/group/each time point. **; p < 0.005.

Recent evidence suggests that IL-10 can reduce BLM-induced lung injury by inhibiting the production of IL-17A and TGF-β [16]. Moreover, previous studies showed that IL-10 was an important element of the counter-regulatory process induced by CpG ODN [24]. Thus, the effect of CpG treatment on pulmonary IL-10 mRNA expression was examined. Extending early findings that examined IL-10 gene expression in the spleen, mice treated with CpG ODN showed slightly increased levels of IL-10 in the lungs 5 days later. When these animals were treated with BLM, IL-10 mRNA levels were significantly higher in the CpG treated vs control groups 3 days later (Figure 6).

**Figure 7:**
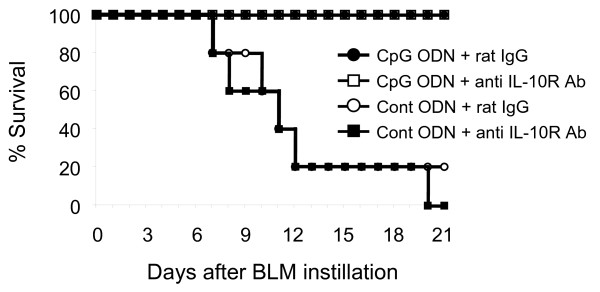
**Effect of anti-IL-10R Abs on CpG induced protection.** Mice were injected i.p. with 200 μg of CpG ODN 5 days before 0.05 U of BLM or PBS (no BLM) was delivery intra-tracheally. Some groups were also treated with 200 μg of anti-IL-10R Abs or isotype matched Abs i.p. on the day of and 5 days after BLM instillation. Results from 5 mice/group are shown.

Further studies examined whether IL-10 alone accounted for the reduced inflammation observed after CpG was administered to BLM treated mice. Initial efforts to utilize IL-10 KO mice failed, as that strain became acutely ill after CpG administration (independent of BLM treatment). Thus, normal mice were injected with neutralizing doses of anti-IL-10 receptor antibodies (anti-IL-10R Abs) immediately prior to and 5 days after BLM administration. Unfortunately, no reduction in CpG dependent protection was observed in those studies (Figure 7).

## Discussion

CpG ODN activate the innate immune system by stimulating cells expressing TLR9. The resultant immune response is short lived, with the up-regulation of mRNA encoding pro-inflammatory cytokines and chemokines generally peaking between 3 - 9 hr. This rapid but short term activity is supported by evidence that intraperitoneal injection of CpG ODN induces pulmonary inflammation lasting from 1 - 6 hr such that no evidence of inflammation is detectable at 5 days [29]. Recent studies indicate that this early pro-inflammatory response is followed by a prolonged period of gene suppression mediated by a counter-regulatory process [24]. Microarray studies of spleen cells showed that the signaling cascade triggered by CpG ODN resulted in the up-regulation of genes including IL-10 whose product suppressed the initial immune response. This down-regulation occurred under physiological conditions, was highly reproducible, and peaked 5 days after CpG administration [24]. Extending those findings, data presented in Figure 6 demonstrates that expression of the IL-10 gene in the lungs is also increased 5 days after CpG administration (Figure 6). The current work is the first to examine whether this counter-regulatory process can be harnessed to prevent the pathological inflammation triggered by a different agent; in this case, the lung injury induced by BLM.

Bleomycin is an important chemotherapeutic agent [1-3]. Unfortunately, the lungs are deficient in the enzyme that detoxifies BLM, and dose-limiting pulmonary toxicity arises in nearly 20% of patients [7,10-12]. BLM generates DNA-cleaving free radicals that injure lung cells, leading to the release factors that attract and stimulate alveolar macrophages to produce a variety of cytokines and chemokines, key among which are IL-1β, IL-6, KC and MIP-2. These factors recruit and activate additional inflammatory cells, culminating in the development of pulmonary fibrosis [13,14].

CpG ODN have historically been used to bolster immune reactivity in animals with established disease (including infection, allergy and cancer). In the context of BLM induced fibrosis, Luckhardt et al. reported that CpG treatment 14 days after intrathecal injection of BLM reduced lung fibrosis [30]. Although the precise mechanism was not shown, they suggested that the enhancement of IFN-β production by CpG ODN might be associated with the reduction of fibrosis. IL-12, which is a representative pro-inflammatory cytokine induced by CpG ODN, was also reported to inhibit BLM induced lung fibrosis. Keane et al. reported that 16 consecutive days of IL-12 administration reduced BLM-induced lung fibrosis and suggested that this protective effect was mediated via IFN-γ [31]. In all previous studies, CpG or IL-12 was administered after BLM and the effect of the resultant Th1 response on lung fibrosis was monitored. There have been several studies examing the effect of administering CpG ODN prior to the development of disease. In an animal model of stroke, administering CpG ODN 3 days before the onset of brain ischemia significantly reduced infarct size and this neuroprotective effect was associated with CpG-induced TNF-α and type I interferon production [32,33]. In a model of LPS-induced lung inflammation, CpG ODN administered 1 - 12 hours before LPS inhibited pulmonary injury in an IL-12 dependent manner [34]. Similarly, CpG ODN delivered shortly before pathogen challenge elicited an inflammatory response that improved host resistance to infection [35,36]. Yet the activity of CpG ODN in these systems was uniformly dependent on the early induction of a pro-inflammatory response. The current work is the first to examine whether the counter-regulatory response peaking 5 days after CpG administration might be harnessed to therapeutic advantage.

Results show that mice pre-treated with CpG ODN are resistant to the pulmonary damage induced by BLM instillation. CpG dependent protection was maximal in mice treated with 200 μg of CpG ODN 5 days prior to BLM instillation (Figure 1). That treatment regime completely prevented the mortality and significantly reduced the acute and chronic pulmonary morbidity elicited by BLM. It also significantly reduced other measures of pulmonary inflammation, including collagen deposition, leukocyte accumulation, and the accumulation of IL-1β, IL-6, KC and MIP-2 in BAL fluid (Figures 2-4). Lowering the dose of CpG ODN also reduced the protection achieved, while substituting control ODN resulted in a complete loss of protection (Figure 1). When administered only 3 days before BLM (the time at which the counter-regulatory response first arose), CpG treatment was less effective, leading to only a 26% improvement in survival (p < 0.05, data not shown). Delaying the initiation of therapy until the day of or after BLM administration worsened the inflammatory process (Figure 1C). These outcomes were not surprising, as CpG ODN have an immediate but short lasting inflammatory effect on the lungs. We conclude that CpG ODN must be administered several days prior to BLM instillation to harness the counter-regulatory process and prevent pulmonary inflammation.

Studies were conducted to clarify the mechanism by which CpG ODN blocked BLM-dependent pulmonary inflammation. Previous work showed that the concentration of IL-17A and TGF-β in BAL rose significantly after the instillation of BLM (Figure 5 and [16-18]), and that these factors supported the development of BIP by promoting the production of pro-inflammatory cytokines and chemokines (including IL-1β, IL-6, KC and MIP-2). Studies using neutralizing Abs and KO mice showed that the elimination of IL-17A and/or TGF-β led to a significant reduction in BLM-induced pulmonary fibrosis [16-18,37]. Other reports showed that IL-10 down-regulated the production of both IL-17A and TGF-β [16] and that IL-10 was a key mediator of the counter-regulatory process responsible for suppressing CpG induced immune activation [23,25,26].

Consistent with those findings, IL-10 mRNA levels rose significantly while the levels of IL-17A and TGF-β1 fell significantly in the BAL of mice pre-treated with CpG DNA (Figures 5, 6). Control ODN had no such effect, establishing the specificity of this effect. However when the activity of IL-10 was blocked with anti-IL-10R Abs, no reduction in CpG mediated protection was observed (Figure 7). These findings are consistent with the interpretation that multiple elements of the counter-regulatory process triggered by CpG ODN contribute to the reduction in BLM-induced pulmonary inflammation, such that eliminating a single component (such as IL-10) does not undermine the protective response.

While failing to identify the precise factors responsible, current results establish the principle that the counter-regulatory process responsible for suppressing TLR9-induced gene activation can be harnessed to block the inflammation arising from an unrelated stimulus. As pulmonary inflammation represents the dose-limiting toxicity of many chemotherapeutic agents, the protective activity of CpG pre-treatment identified herein may be of widespread utility. In this context, since chemotherapy schedules are typically established weeks in advance, the administration of CpG ODN prior to BLM (or other agents) could be readily incorporated into these therapeutic protocols.

## Competing interests

Dr. Klinman and members of his lab hold or have applied for patents involving the use of immunomodulatory oligonucleotides. The rights to all such patents have been assigned to the Federal Government.

## Authors’ contributions

TK and KT performed the experiments. DCH performed the histologic analyses. TK and DMK contributed to the interpretation of the data and wrote the manuscript. All authors read and approved the final manuscript.
